# An Energy Metabolism Study on the Efficacy of Naoxintong Capsules against Myocardial Infarction in a Rat Model

**DOI:** 10.1155/2022/3712500

**Published:** 2022-07-23

**Authors:** Jie Zhao, Yi Ouyang, Huanhuan Wang, Huaqing Lai, Shaowei Hu, Liying Tang, Hongjun Yang, Hongwei Wu

**Affiliations:** ^1^Institute of Chinese Materia Medica, China Academy of Chinese Medical Sciences, Beijing, China 100700; ^2^Zunyi Medical University, Zunyi, China 563000; ^3^Medical Experimental Center of Chinese Academy of Traditional Chinese Medicine, Beijing, China 100700

## Abstract

**Background:**

In myocardial ischemia, optimizing the myocardial metabolic phenotype to improve cardiac function is critical. Naoxintong capsules (NXT) are widely prescribed in Chinese medicine for the treatment of cerebrovascular and cardiovascular diseases.

**Methods:**

In this study, a rat model of myocardial infarction was established by ligation of the left anterior descending coronary artery. The structure and function of the heart were evaluated using echocardiography. The pathological changes of the rat myocardium and the myocardial volume collagen fraction (CVF) were examined using hematoxylin-eosin (HE) and Masson's trichrome staining (Masson). The expression of TNF-*α* and IL-6 were detected by immunohistochemistry. The level of cTnT was also measured to evaluate myocardial injury. In order to study the changes in energy metabolism in myocardial infarction and the effects of NXT, a targeted analysis method for detecting the 29 energy metabolites in cardiac muscle tissue was developed based on UPLC-QQQ-MS. Western blotting was used to detect the expression of proteins related to energy metabolism in myocardia.

**Results:**

In the rat model of myocardial infarction, NXT showed obvious effects, such as improving heart function and increasing LVEF and LVFS. HE staining, Masson staining, and immunohistochemical results revealed that NXT decreased inflammatory infiltration, improved myocardial fibrosis, and reduced infarct size. In addition, NXT significantly reduced the level of serum cTnT. The levels of the 29 energy metabolites in cardiac muscle tissue were analyzed using a newly developed targeted analysis method. Compared to the sham group, the levels of 17 metabolites from different energy metabolic pathways, including four compounds in glycolysis metabolism, four compounds in TCA cycle, three compounds in oxidative phosphorylation, four compounds in purine metabolism, and two compounds in glutathione metabolism, displayed obvious changes induced by myocardial ischemia. Expressions of SIRT1, PGC-1*α*, and ATP5D proteins related to energy metabolism were decreased after myocardial infarction. These perturbations could all be reversed by NXT intervention, suggesting that the therapeutic effects of NXT were partially due to interferences with energy metabolisms.

**Conclusion:**

This study provides a useful approach for investigating the mechanism of myocardial infarction and evaluating the efficacy of NXT from energy metabolism.

## 1. Introduction

Myocardial infarction is the leading cause of death and disability worldwide [1]. It is a process of myocardial remodeling that includes cardiomyocyte hypertrophy or apoptosis, myocardial fibrosis, a series of inflammatory reactions, and the formation of new blood vessels [2]. The damage caused by myocardial infarction eventually leads to irreversible heart failure [3].

Cardiovascular disease is a complex and intractable disease. In long-term clinical practice, the use of multiple compounds in traditional Chinese medicine (TCM) to treat cardiovascular disease has comprehensive medical effects and fewer side effects compared to traditional/chemical medicines [4, 5]. NXT, a Chinese patent medicine recorded in the Chinese Pharmacopoeia (Chinese Pharmacopoeia Commission, 2020), has been used for the treatment of cardiovascular and cerebrovascular diseases by improving clinical symptoms, such as chest pain, palpitation, shortness of breath, and hemiplegia [6, 7]. It is derived from a classic TCM prescription named Bu–Yang–Huan–Wu–Tang and contains 16 Chinese herbal and animal medicines, including *Astragalus membranaceus* var. mongholicu (Bunge) P.K. Hsiao., root; *Angelica sinensis* (Oliv.) Diels., root; *Salvia miltiorrhiza* Bunge., root and rhizome; *Paeonia lactiflora* Pall., root; *Ligusticum striatum* DC., root and rhizome; *Prunus davidiana* (Carr.) Franch., seeds; *Carthamus tinctorius* L., flowers; *Boswellia sacra* Flueck., resin; *Commiphora myrrha* (Nees) Engl., resin; *Spatholobus suberectus* Dunn., rattans; *Achyranthes bidentata* Blume., root; *Cinnamomum cassia* (L.) J. Presl., twigs; Morus alba L., twigs; *Pheretima aspergillum* (E. Perrier)., body; *Buthus martensii* Karsch., body; and *Hirudo nipponica* Whitman., body.

In our previous studies, we established a chemical profile for NXT using ultra-high-pressure liquid chromatography coupled with linear ion trap-Orbitrap tandem mass spectrometry and identified or tentatively characterized a total of 178 components in NXT [8]. Furthermore, as a standardized product, a few components of NXT, including hydroxyl safflor yellow A, paeoniflorin, ferulicacid, salvianolic acid B, and ligustilide, have been quantitatively analyzed using HPLC-UV for quality control. According to other studies, NXT has been proved to be widely used and plays a cardioprotective role in various cardiovascular diseases such as coronary artery/heart disease (CAD/CHD), congenital heart disease, rheumatic heart disease, diseases of the aorta and arteries, cardiomyopathies, and cardiac arrhythmias [9]. NXT can exert a variety of pharmacological effects. In a carrageenan-induced thrombosis mouse model, it has been reported that NXT can substantially reduce thrombi and inflammatory factors such as TNF-*α* [10]. A vitro study shows that NXT counteracts oxidative stress injury induced by H_2_O_2_ in H9c2 cardiomyocytes [11]. In a MCAO rat model, NXT can reduce capase-3/9 expression, suggesting its antiapoptotic effect of NXT [12].

It has been established that in a healthy non-ischemic heart, 60-90% of the energy comes from *β*-oxidation of fatty acids, with the remaining 10-40% coming from carbohydrate metabolism, including glycolysis, lactate oxidation, and tricarboxylic acid (TCA) [13–15]. Optimizing myocardial metabolic phenotype to improve cardiac function after myocardial ischemia without causing any direct negative hemodynamic or inotropic effects is considered to be an attractive therapeutic approach [16].

In our previous studies, we examined the effects of using NXT to treat cerebral ischemia using an untargeted metabolomics technology. Parts of the identified biomarkers of NXT action on cerebral ischemia were specifically related to disturbances in energy metabolism [17, 18]. However, the therapeutic effects of NXT and its mechanisms in the treatment of myocardial infarction are still not well understood. Furthermore, untargeted metabolomics analysis is a relative quantitative approach with lower sensitivity and accuracy for certain types of metabolites compared to targeted metabolomics technology. Therefore, biomarkers discovered through an untargeted metabolomics approach require further validation.

In this study, a targeted metabonomic approach based on UPLC-QQQ-MS was developed and used to absolutely quantify 29 metabolites in the myocardial tissue in order to investigate the mechanism of NXT in improving myocardial infarction from the perspective of energy metabolism. The 29 identified metabolites were involved in biological processes of energy metabolism, including glycolysis, the TCA cycle, oxidative phosphorylation (OXPHOS), purine metabolism, and glutathione metabolism. The comprehensive and acute analysis of the energy metabolic markers in this study may provide a better understanding of the complex pathogenesis of the myocardial ischemia metabolic phenotype and the potential mechanism of NXT in vivo.

## 2. Materials and Methods

### 2.1. Animals and Reagents

Male Sprague-Dawley rats were housed in an animal room at a temperature of 25°C and relative humidity of 50%, with a 12-hour light/dark cycle. All animals were acclimatized for one week prior to surgery. The animals had free access to water and food (Beijing Keaoxieli Co, Ltd.). All experimental animal procedures were performed in accordance with the National Institutes of Health guide for the care and use of Laboratory animals (NIH Publications No. 8023, revised 1978) and reviewed by the Ethics Committee of China Academy of Chinese Medical Science. A total of 29 energy metabolite standards, such as alpha-ketoglutarate, oxaloacetate, succinate, phosphoenolpyruvate, L-malic acid, lactate, cis-aconitate, GMP, NAD, NADPH, NADP, ADP, adenosine monophosphate, cyclic AMP, isocitrate, citrate, pyruvate, fumarate, adenosine 5′-triphosphate (ATP), guanosine 5′-diphosphate (GDP), guanosine 5′-triphosphate (GTP), thiamine pyrophosphate (TPP), flavin mononucleotide (FMN), D-fructose 1,6-bisphosphate, beta-D-fructose 6-phosphate, 3-phospho-D-glycerate, D-glucose 6-phosphate, dihydroxyacetone phosphate, and acetyl coenzyme A (acetyl-CoA) (purity >98%, all), were all purchased from Sigma-Aldrich (St. Louis, MO, USA). Succinic acid-d6 (internal standard (IS)) was purchased from Cambridge Isotope Laboratories, Massachusetts, USA (DLM-831-5, CAS 21668-90-6). Acetonitrile and methanol were purchased from Fisher Scientific (Shanghai, China). All chemicals used in this study were of HPLC grade unless stated otherwise. NXT (batch number: 140156) was purchased from Buchang Pharma Co. Ltd, Shanxi Province, China.

### 2.2. Surgery and Drug Administration

As previously mentioned, a rat coronary artery ligation model was established. In brief, rats were anesthetized with 1% sodium pentobarbital (0.5 ml/100 g) and intubated using a Zoovent ventilator (ALC-V8S, Shanghai Alcott Biotechnology Co., Ltd.). After vertical thoracotomy and pericardectomy, the heart was exposed, and a 5-0 silk suture was used to ligate LAD approximately 2-3 mm from its starting point to induce myocardial infarction.

A total of 50 rats were randomly divided into sham (*n* = 10) and myocardial ischemia (*n* = 40) groups. All rats with coronary artery ligation were randomly allocated to treatment groups (NXT-H, *n* = 10; NXT-L, *n* = 10; and valsartan, *n* = 10) and model group (*n* = 10). Rats were then administrated with 1000 mg/kg NXT (per day, named NXT-H group), 250 mg/kg NXT (per day, named NXT-L group), 8 mg/kg valsartan (per day, named Val group), and saline (model group and sham group) orally for two weeks.

### 2.3. Echocardiography

Two weeks after modeling, echocardiography was performed on all animals using a Visual Sonics Vevo 770 (Toronto, Canada) with a 17.4 MHz central frequency scan at various time points after surgery. During echocardiography, rats were anesthetized with oxygen and isoflurane (64% N_2_, 32% O_2_, and 4% isoflurane), and freely breathing animals were maintained with oxygen and isoflurane (1.75%). Long axis cine loops and M-mode images of the LV and atrium were acquired in the parasternal long-axis. The left ventricular ejection fraction (LVEF; %) and left ventricular fractional shortening (LVFS; %) were measured off-line according to the guidelines of the Vevo 770 system software.

### 2.4. Sample Collection and Preparation

After echocardiography, blood was taken from the abdominal aorta of the rats, and their entire bodies were perfused with saline from the heart. Finally, the heart was removed, with the right side-striped and the remaining left side divided into three parts. Penumbral tissue was reserved for targeted metabolomics analysis. Both tissue and serum samples were stored at −80 °C for further analysis.

### 2.5. Histology Staining and Determination of Serum cTnT Level

Paraffin tissue section slides with a thickness of 5 *μ*m were used for histology staining. After deparaffinization, the slides were stained with hematoxylin and eosin (HE) and Masson's trichrome stain according to the instructions.

The levels of cTnT were determined using ELISA kits (product code: E-EL-R0151c; Elabscience, China) according to the manufacturer's instructions.

### 2.6. Immunohistochemical Staining of Heart Tissue

Paraffin sections of myocardial tissue were rehydrated with H_2_O_2_ after dewaxing. Sections were loaded with primary antibodies overnight. Secondary antibody incubation was followed by incubation for 10 min with streptavidin peroxidase. DAB (1×) was applied as the chromogen to incubate with the sections for 10 min at room temperature and then dyed by hematoxylin counterstain. Photos were captured under a light microscope and counted using ImageJ software. The antibodies used were as follows: IL-6 (E-AB-40073, Elabscience) and TNF-*α* (ABM0066, Abbkine).

### 2.7. Analysis of Targeted Energy Metabolites by UPLC-QQQ-MS

#### 2.7.1. Sample Preparation

The sham, model, and NXT-H groups were selected for targeted analysis. Briefly, 60 mg of the cardiac muscle tissue was weighed; 100 ul water homogenate was added and vortexed for 60 s; then, 400 ul methanol acetonitrile solution (1 : 1, v/v) and 10 *μ*L (10 mmol/L) succinic acid -d6 (IS) were added and vortexed for 60 s. Following that, the samples were sonicated for 30 min at low temperature twice and stored at −20 °C for 1 h to precipitate the protein. The supernatant was centrifuged for freeze-drying and the sample was stored at −80 °C. When performing tests, 100 *μ*L methanol was added for detection.

#### 2.7.2. Chromatographic and Mass Spectrometric Conditions

The samples were separated using the Agilent 1290 Infinity LC ultra-high performance liquid chromatography system. The mobile phase consisted of solvent A (water containing 10 mM ammonium acetate) and solvent B (acetonitrile) with gradient elution (90-40% B at 0-18 min, 40-90% B at 18-18.1 min, and 90-90% B at 18.1-23 min). Chromatographic separation was carried out on a Waters ACQUITY UPLC BEH Amide (1.7 *μ*m, 2.1 mm × 100 mm column) designed to retain polar analytes that are too polar for reversed-phase chromatography. Samples were placed in a 4 °C autosampler, with the column temperature set to 45 °C, the flow rate set to 0.3 mL/min, and the injection volume set to 2 *μ*L. A quality control (QC) sample prepared according to the standards was set for every specific number of experimental samples in the sample queue to detect and evaluate the stability and repeatability of the system.

The 5500 Q TRAP mass spectrometer (AB SCIEX) was used for mass spectrometry analysis. The electrospray ionization source (ESI source) was in the negative ion mode with the following conditions: source temperature at 450 °C, ion Source Gas1 (Gas1) at 45, Ion Source Gas2 (Gas2) at 45, Curtain gas (CUR) at 30, and Ion Sapary Voltage Floating (ISVF) at −4500 V. Quantification was performed for all the detected compounds using multiple reaction monitoring (MRM) based on negative ion scanning. The ion transition and collision energies of the precursor product are shown in Table 1.

#### 2.7.3. Method Validation

*(1) Linearity, Sensitivity, and Carryover*. Individual standards (~5 mg) were prepared by dissolving the solids in 5 mL of distilled water. A series of standards ranging from 1 to 1000 ng mL^−1^ was prepared by serial dilution with the initial mobile phase. Before injection, 90 *μ*L of each standard was mixed with 10 *μ*L of succinic acid-d6 (IS, 10 mm/L). The series of standards was used to plot calibration curves. Sensitivity was evaluated using limits of detection (LOD) and limits of quantification (LOQ), with the corresponding standard solution at a signal-to-noise (S/N) ratio of ~3 and ~10, respectively. In this study, carryover was investigated due to the wide concentration range of quantitation. Carryover was performed by injecting a blank vehicle sample after injecting the standards with an upper limit of quantitation concentrations.

*(2) Precision Stability and Repeatability*. The precision of the LC/MS system was evaluated using QC samples. Stability was tested using QC samples at 4 °C and analyzed at 0, 2, 4, 8, 12, and 24 h. The relative standard deviation (RSD) of peak area was used as a measure of precision and stability. Six samples were prepared in parallel and tested. The RSD of the compound content was obtained to determine repeatability.

### 2.8. Western Blotting

The heart tissue was cut into small fragments, and protein extraction was performed according to the instruction of Protein Assay Kit (P0013, Beyotime, China). After separated on a 10% SDS-PAGE (P0014, Beyotime, China), the protein samples were transferred to a polyvinylidene fluoride membrane (Millipore, IPVH00010), which was blocked with bovine serum albumin (BSA). After that, the primary antibody specific for PGC-1*α* (66639-1, Proteintech Group, China) or SIRT1 (60303-1, Proteintech Group, China) or ATP5D (14893-1, Proteintech Group, China) was added to the membranes and incubated for 24 h at 4 °C. The second antibody goat anti-rabbit IgG (H + L) (Jackson, 111-035-003) was loaded, and the signal of the proteins was detected by scanning densitometry.

### 2.9. Statistical Analysis

The statistical analyses were conducted through the use of a one-way ANOVA followed by Tukey multiple comparison. The data are expressed as mean ± standard deviation. *P* < 0.05 means that the difference between tested groups is statistically significant. Principal component analysis (PCA) was performed using the Simca-P 14.1 software. The GraphPad Prism 9.0 software was used for visualization.

## 3. Results

### 3.1. NXT Improves the Cardiac Function of Rats with Myocardial Infarction

**Figure 1:**
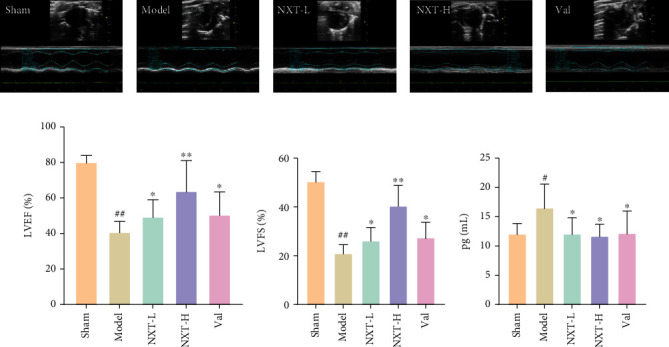
Effects of NXT on rat cardiac function. (a) Echocardiograms in different rat groups. (b) LVEF in different rat groups. (c) LVFS in different rat groups. (d) The levels of cTnT in the serum of the different rat groups. ^##^*P* < 0.01, ^#^*P* < 0.01, model group vs. sham group; ^∗∗^*P* < 0.01, ^∗^*P* < 0.05, treatment group vs. model group.

In the sham group, the heart contour and the ventricular wall systolic/diastolic rhythm were normal, as seen in Figure 1(a). In the model group, the whole heart cavity was enlarged, and the thickness of the myocardium at the infarcted area was thinner and bulged out of the heart contour. Compared to the model group, the contraction amplitude of the left ventricle was significantly enhanced in the administration groups. The NXT-L and Val groups had similar effects on the recovery of ventricular contraction rhythm, while the NXT-H group had the best effect. Other detailed parameters of echocardiography are shown in Table 1.

In order to investigate the effect of NXT on myocardial infarction in rats, LVEF and LVFS were measured using cardiac echography. As shown in Figures 1(b) and 1(c), EF and FS decreased significantly after myocardial infarction compared to the sham operation group (*P* < 0.05), indicating heart dysfunction. Both the low- and high-dose NXT administration groups significantly improved LVEF and LVFS compared to the model group (*P* < 0.05), indicating that NXT administration effectively protected the heart against myocardial infarction.

As shown in Figure 1(d), the level of cTnT was significantly elevated compared to the sham group (*P* < 0.05), as determined by ELISA. However, after drug intervention, the cTnT content of the NXT and Val groups significantly decreased, indicating that both NXT and Val could effectively alleviate myocardial damage in rats after myocardial infarction. Based on the evaluation of cardiac function, ventricular structure indicators, and myocardial enzymes, the NXT-H group demonstrated significant improvements.

### 3.2. NXT Inhibits Cardiac Structural Remodeling after MI

**Figure 2:**
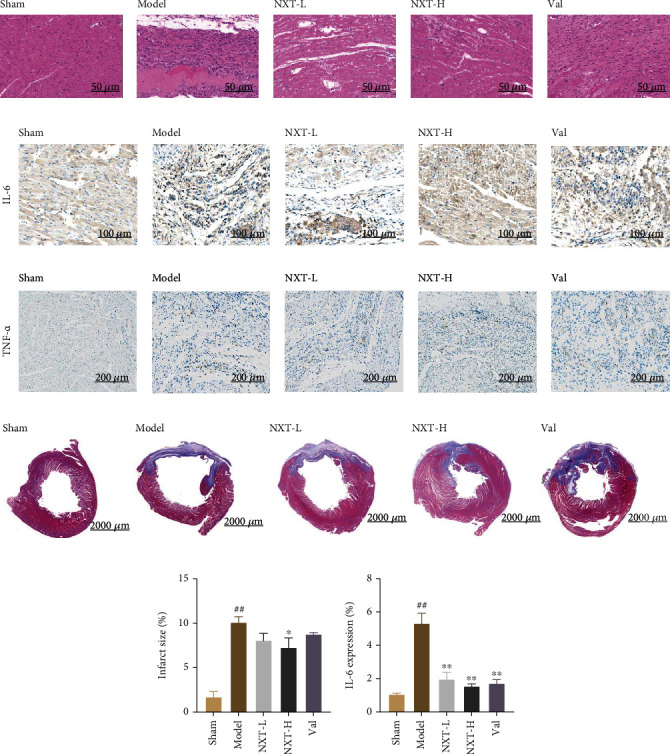
Effect of NXT on the left ventricular remodeling of the ischemic myocardium. (a) HE staining of myocardial tissue of each rat group (20×). (b) Immunohistochemical staining for IL-6 and TNF-*α*. (c) Masson staining of myocardial tissue of each rat group (0.6×). The blue stain indicates infarct area (collagen); the red stain indicates myocardial fibers. (d) Quantification of the infarct size using the equation: infarct size (%) = area of collagen staining as an indicator of myocardial infarction/total area of the left ventricle∗100. (e) Quantified immunohistochemical staining of IL-6. ^##^*P* < 0.01, ^#^*P* < 0.01, model group vs. sham group; ^∗∗^*P* < 0.01, ^∗^*P* < 0.05, treated group vs. model group.

The HE staining results revealed that the myocardial tissues in the sham operation group were tightly arranged and that the morphology of the myocardial cells was intact (Figure 2(a)). In the model group, myocardial tissue was irregularly distributed, the structure was disordered, the morphology of myocardial cells was altered, the myocardial space was significantly enlarged, and there was infiltration of inflammatory cells. In the NXT groups and Val group, myocardial tissue injury improved to a certain extent, myocardial tissue arrangement was denser, and inflammatory infiltration was not obvious. To further investigate the effect of NXT on inflammatory infiltration after myocardial infarction, the expressions of TNF-*α* and IL-6 were evaluated by immunohistochemistry. The expression of TNF was lower in each group, and the difference was not significant. However, as shown in Figures 2(b) and 2(e), in the model group, the expression IL-6 was significantly higher than that of the sham group. After drug intervention, the expressions of IL-6 in both NXT groups and Val group were decreased.

The Masson staining results showed that the morphology of cardiomyocytes in the sham operation group was normal, with no collagen deposition, and the arrangement of muscle fibers was dense and regular (Figures 2(c) and 2(d)). In the model group, a large amount of collagen was deposited, myocardial fibrosis tissue increased, and the infarct area increased. Compared to the model group, myocardial injury in the treatment groups was improved to a certain extent, there was a small amount of collagen deposition, and the infarct size and degree of myocardial fibrosis were both reduced.

These findings indicated that NXT can decrease inflammatory infiltration, improve myocardial fibrosis, and reduce infarct size in rats with myocardial infarction.

### 3.3. Targeted Metabonomic Analysis for Energy Metabolites by UPLC-QQQ-MS

#### 3.3.1. Method Validation

**Figure 3:**
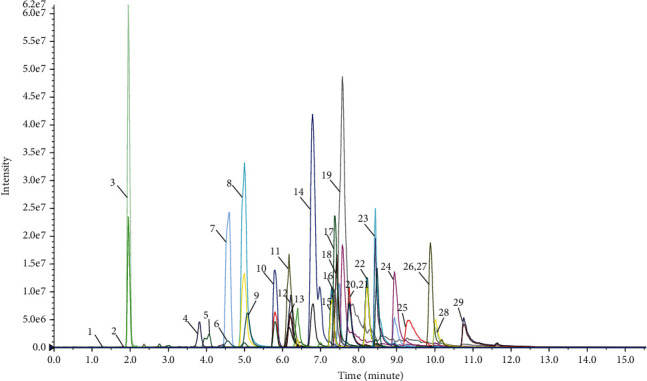
A typical overlap of a multiple reaction monitoring (MRM) chromatogram of the 29 detected metabolites.

A targeted metabonomic analysis method for accurately detecting the 29 energy metabolites in the cardiac muscle tissue was developed and validated by UPLC-QQQ-MS in a pre-experiment.  Figure 3 depicts a typical overlap of a multiple reaction monitoring (MRM) chromatogram of the 29 detected compounds.

The linear response of the calibration curves was determined using a series of standards. As shown in Table 1, a correlation coefficient (*r*) for all analytes ranged from 0.9821 to 0.9996, indicating that the regression model fitted the respective concentration ranges well. Furthermore, the method exhibited excellent sensitivity, as measured by the signal-to-noise (S/N) ratio. The LOD and LOQ for all analytes were in the range of 0.03–8 umol/L (at S/N > 3) and 0.1-25 umol/L (S/N > 10), respectively. For the carryover evaluation, the blank vehicle sample injected following the upper limit of quantitation standard injection had no significant peaks at all analyte retention times. Since the carryover measurement can be affected by its position in the sampling sequence due to adsorptive carryover issues, blank vehicle samples were injected at regular intervals (every ten samples) throughout the analytical run.

In addition, the ion pairs detected by MRM, including parent and daughter ions of the analytes, were all different and are listed in Table 2. There was no observed significant crosstalk between IS and analytes using this method.

The precision of the LC/MS system was evaluated using QC samples prepared from the standards. Six quality control (QC) samples were run at the beginning of the sequence. Furthermore, the QC samples were run at regular intervals (every ten samples) throughout the entire sequence. The RSDs of the peak areas of all 29 compounds were determined, and all were found to be less than 10%, which was consistent with the criteria for biological sample analysis.

The stability of all analytes was evaluated by analyzing one prepared sample, while the samples were stored at 4 °C before the injection. The RSDs of the peak areas of the analytes were within 5%, suggesting that the sample was stable within 24 h of injection.

In order to confirm repeatability, six samples prepared in parallel were analyzed, and the RSDs of the peak areas of the analytes in the detected metabolites were all less than 10%. Taken together, the above results indicated that this analytical method is accurate, stable, and reproducible and can be used to analyze energy metabolites in cardiac muscle tissue.

### 3.4. Changes in Energy Metabolomics Features in the AMI Rat Model Induced by NXT

In this study, 29 metabolites related to energy metabolism were identified, including glycolysis, TCA cycle, oxidative phosphorylation, purine metabolism, and glutathione metabolism. During glycolysis, the following eight metabolites were detected: phosphoenolpyruvate, lactate, pyruvate, D-fructose 1,6-bisphosphate, beta-D-fructose-6-phosphate, 3-phospho-D-glycerate, D-glucose 6-phosphate, and dihydroxyacetone phosphate. In the TCA cycle, the following ten metabolites were detected: alpha-ketoglutarate, oxaloacetate, succinate, L-malic acid, cis-aconitate, isocitrate, citrate, fumarate, thiamine pyrophosphate (TPP), and acetyl coenzyme A (acetyl-CoA). During oxidative phosphorylation, the following four compounds were detected: NAD, flavin mononucleotide (FMN), ADP, and ATP. The five metabolites involved in purine metabolism were as follows: GMP, adenosine monophosphate, cyclic AMP, guanosine 5′-diphosphate (GDP), and guanosine 5′-triphosphate (GTP). The two metabolites detected during glutathione metabolism were NADPH and NADP.

**Figure 4:**
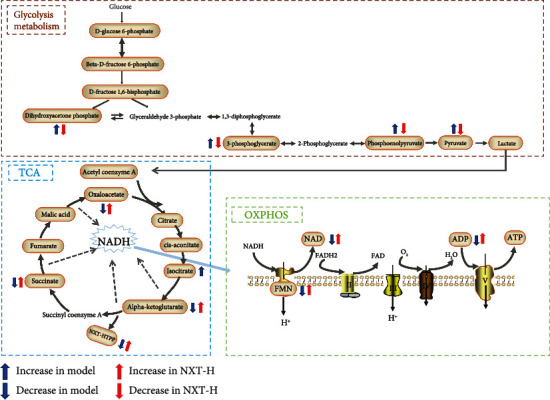
Schematic diagram of the energy circulation and changes in differential metabolites after myocardial ischemia and NXT intervention. The metabolites in brown border are the metabolites detected in this study.

The main metabolic processes of the substances, such as glycolysis, TCA cycle, and oxidative phosphorylation, are shown in Figure 4. The changes in the content of various metabolites in the metabolic processes after myocardial infarction and after the NXT intervention are illustrated.

**Figure 5:**
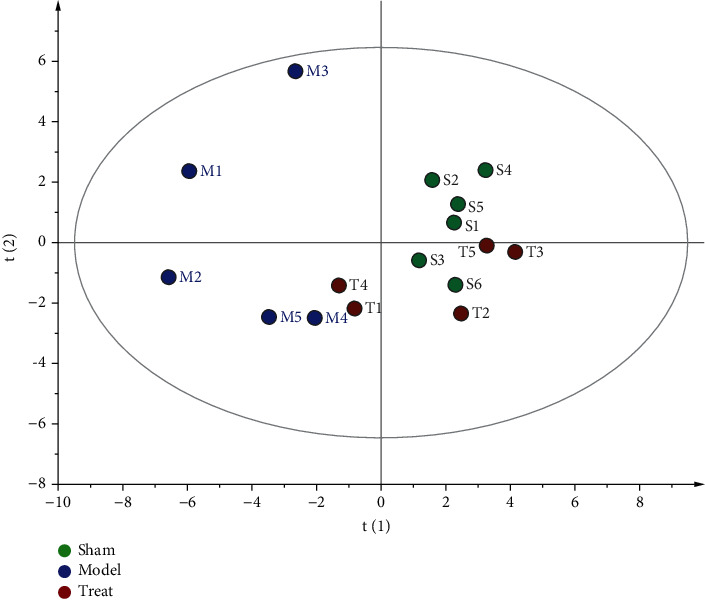
The PCA scores plot based on the contents of all the detected metabolites.

In order to investigate the global metabolism variations of all the detected energy metabolites, PCA was first used to evaluate the observations by the SIMCA software (version 13.0). PCA, an unsupervised pattern recognition method for handling metabonomic data, is capable of classifying the energy metabolic phenotypes based on all imported samples. An overview of all samples is shown in the PCA score plot (cf.  Figure 5). In general, a grouping trend was observed, especially for the model and sham groups (R2X, 0.503; Q2, 0.398). Although the sample distributions of the model group were relatively scattered, they can be clearly distinguished from the sham group. This finding indicated that myocardial infarction disrupted the metabolism of endogenous substances when compared to the normal state. Furthermore, the PCA scores plot revealed an effect on the myocardial infarction-induced changes in energy metabolite levels after NXT intervention, despite the trajectory of the NXT groups not being restored to normal levels (i.e., sham groups).

#### 3.4.1. Glycolysis Metabolism

Under normal aerobic conditions, most of the energy required by the heart comes from fatty acid oxidation, with the rest coming from carbohydrates, such as glucose [19]. However, because oxygen supply is limited during myocardial ischemia, fatty acid and carbohydrate oxidation, as well as ATP production, are reduced. At this stage, glycolysis becomes a more important source of energy [20].

Glycolysis is the metabolic pathway that converts glucose into pyruvate. The free energy released during this process is used to generate high-energy compounds ATP and NADH. Glycolysis is the first step in the process of sugar metabolism, which consists of ten enzymatic reaction steps that ultimately result in pyruvate formation. Under aerobic conditions, acetyl coenzyme A is produced and enters the TCA cycle. During myocardial ischemia, the increased glycolytic activity causes lactic acid accumulation, leading to H^+^ accumulation, Ca^+^ overload, and a decrease in myocardial contractility [21].

**Figure 6:**
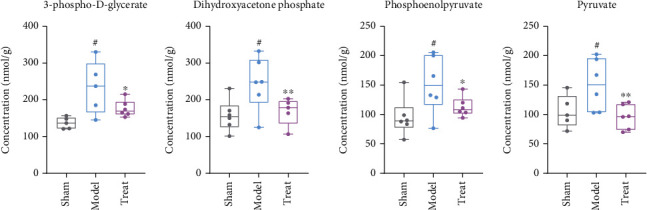
The detected metabolites in glycolysis metabolism, with significant differences between the model, sham, and treated groups. ^##^*P* < 0.01, ^#^*P* < 0.01, model group vs. sham group; ∗∗*P* < 0.01, ∗*P* < 0.05, treated group vs. model group.

In this study, eight metabolites were detected during glycolysis: glucose-6 phosphate, fructose-6 phosphate, 1,6-fructose diphosphate, dihydroxyacetone phosphate, 3-phospho-D-glycerate, phosphoenolpyruvate, pyruvate, and lactate, as shown in Figure 5. Among them, dihydroxyacetone phosphate, 3-phospho-D-glycerate, pyruvate, and phosphoenolpyruvate were significantly increased in the model group compared to the sham operation group (*P* < 0.05), indicating an increase in glycolysis metabolism. Furthermore, after NXT treatment, all four metabolites had an obvious callback trend (*P* < 0.05), suggesting that glycolysis metabolism was inhibited (Figure 6).

Notably, partial inhibitors of myocardial fatty acid oxidation have been shown to reduce ischemic dysfunction and tissue damage in animal models of ischemia and reperfusion and are beneficial to patients with chronic stable angina in clinical trials. These effects are attributed to increased pyruvate oxidation, decreased lactate accumulation and efflux, and reduced proton accumulation. Our findings revealed significantly decreased levels of pyruvate and phosphoenolpyruvate in the NXT treatment group. This implied that as pyruvate levels decrease, NXT increased pyruvate oxidation, thereby providing a protective effect.

#### 3.4.2. TCA Metabolism

Free fatty acids produce less ATP than carbohydrates when exposed to a similar amount of oxygen. Therefore, experimental and clinical data have shown that the transfer of energy substrate metabolism from fatty acid metabolism to glucose is effective for treating acute myocardial infarction [22–24]. The TCA cycle is the main metabolic pathway of glucose used to produce ATP through aerobic respiration. Enzymes involved in the TCA cycle oxidize glucose to produce acetyl-CoA (Acetyl-CoA), which then produces NADH, FADH2, and GTP. These molecules are then used by the oxidative phosphorylation system to produce ATP. In this study, ten metabolites were detected during the TCA process: acetyl coenzyme A, citrate, cis-aconitate, isocitrate, alpha-ketoglutarate, succinate, fumarate, L-Malic acid, thiamine pyrophosphate, and oxaloacetate.

**Figure 7:**
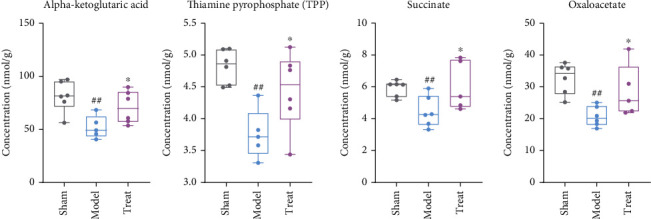
The detected metabolites in TCA, with significantly changes between the model, sham, and treated group. ^##^*P* < 0.01, ^#^*P* < 0.01, model group vs. sham group; ∗∗*P* < 0.01, ∗*P* < 0.05, treated group vs. model group.

After myocardial infarction, we discovered that acetyl coenzyme A, cis-aconitate, and isocitrate showed an increasing trend but there was no significant difference. As shown in Figure 7, the alpha-ketoglutarate, thiamine pyrophosphate, and oxaloacetate were significantly decreased in the model group compared to the sham group (*P* < 0.05). The regulatory enzymes involved in the production of these changed metabolites, such as malic dehydrogenase and isocitrate dehydrogenase, may become potential drug targets. After NXT intervention, the content of the three metabolites showed an increasing trend compared to normal levels (*P* < 0.05), indicating that NXT played a role in the regulation of energy metabolism by TCA cycle.

#### 3.4.3. OXPHOS Metabolism

Oxidative phosphorylation, which occurs in the mitochondria, is the final step in cellular respiration. The lactate and pyruvate oxidation, fatty acid *β*-oxidation, and the citric acid cycle can deliver electrons to the electron transport chain, resulting in ATP formation via oxidative phosphorylation. Almost all aerobic organisms use TCA cycle-oxidative phosphorylation as the main process to produce ATP due to its efficiency in releasing energy [25, 26]. During ischemia, hypoxia and nutrient deficiency lead to many changes in myocardial biochemistry and metabolism. Studies have shown that oxidative phosphorylation ceases during hypoxia, resulting in depolarization of mitochondrial membranes, depletion of ATP, and decreased myocardial contractility [27]. Therefore, understanding the changes in metabolites associated with oxidative phosphorylation under myocardial ischemic conditions will help in optimizing the myocardial metabolic phenotype to improve cardiac function.

**Figure 8:**
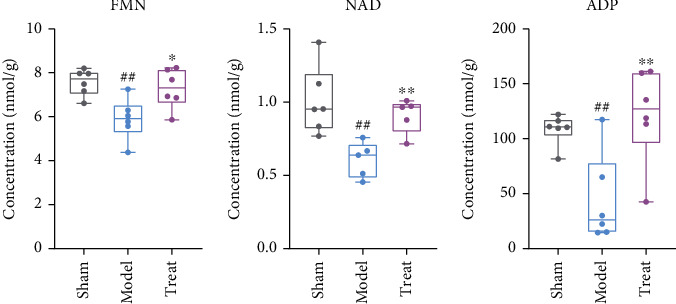
The detected metabolites in OXPHOS, with significantly changes between the model, sham, and treated groups. ^##^*P* < 0.01, ^#^*P* < 0.01, model group vs. sham group; ∗∗*P* < 0.01, ∗*P* < 0.05, treated group vs. model group.

In this study, the content of flavin mononucleotide (FMN), NAD, fumarate, succinate, ATP, and ADP related to oxidative phosphorylation were detected. All these metabolites all showed a downward trend, indicating insufficient energy production under myocardial ischemia conditions. As shown in Figure 8, FMN, succinate, and ADP were found to be significantly reduced. However, after NXT intervention, these three metabolites were significantly restored. This suggested that NXT played a role in improving energy metabolism after myocardial ischemia by enhancing the metabolic activity of oxidative phosphorylation.

#### 3.4.4. The Others

In addition to the above metabolites, GMP, AMP, cyclic AMP, GDP, GTP, NADPH, and NADP were also detected. The first five metabolites are involved in purine metabolism, while the latter two are closely related to glutathione metabolism. NADP and NADPH are cofactors that are involved in the transfer and reservation of reduction potential [28, 29]. In addition, NADPH is an essential electron donor that transfers and reserves reduction potential for numerous anabolic reactions. It is reduced to form NADH by enzymatic reaction, participates in the process of oxidative phosphorylation, and provides power for redox defense [30].

**Figure 9:**
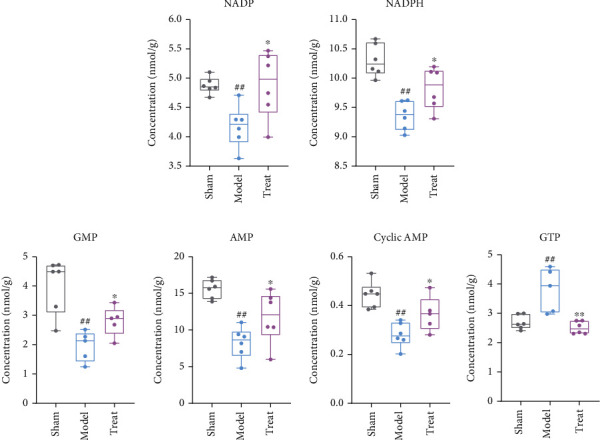
The detected metabolites in (a) glutathione metabolism and (b) purine metabolism with significant changes between the model, sham, and treated groups. ^##^*P* < 0.01, ^#^*P* < 0.01, model group vs. sham group; ∗∗*P* < 0.01, ∗*P* < 0.05, treated group vs. model group.

In this study, NADPH and NADP, which are related to glutathione metabolism, were significantly decreased after myocardial infarction (*P* < 0.05) (Figure 9(a)). Furthermore, after NXT treatment, the levels of both metabolites displayed an apparent tendency to return to normal concentration levels. In purine metabolism, except for GTP, the four metabolites (GMP, AMP, cyclic AMP, and GDP) detected in the model group were significantly decreased compared to the sham group (Figure 9(b)). After NXT intervention, all four metabolites were significantly restored (*P* < 0.05).

According to the above analysis, multiple metabolic pathways of energy metabolism in myocardial tissue were altered after myocardial ischemia. Furthermore, NXT could treat myocardial ischemia by regulating multiple metabolic pathways of energy metabolism, mainly inhibiting glycolysis metabolism and enhancing the metabolic activity of TCA and oxidative phosphorylation.

#### 3.4.5. NXT Enhances the Expression of Energy Metabolism-Related Proteins

Peroxisome proliferator-activated receptor gamma coactivator 1*α* (PGC-1*α*) as an important transcriptional co-activator is not only a key regulator of mitochondrial biosynthesis, but also an important pathway for energy metabolism remodeling [31]. Silent information regulator 1 (SIRT1) is a transcriptional regulator with histone deacetylase activity, which can sense the energy status of the body, regulate the expression of other transcription factors, and also participate in the process of regulating cellular metabolic homeostasis [32]. SIRT1 promotes the deacetylation of lysine residues of PGC-1*α*, initiates the transcriptional activity of PGC-1*α*, and regulates its expression, which further regulates cellular energy metabolism and mitochondrial biosynthesis and protects cardiomyocytes from oxidative stress [33]. ATP5D is a subunit of ATP synthase involved in oxidative phosphorylation. Low expression of ATP5D indicates a decrease in ATP synthesis and a decrease in the body's energy production. Studies have shown that ATP5D can play a protective role against cardiac ischemia-reperfusion injury as a potential drug for preventing energy metabolism disorders [34].

**Figure 10:**
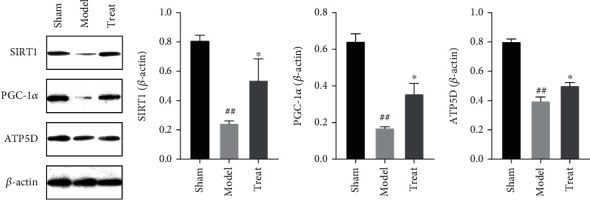
Western blot and statistical analysis of SIRT1, PGC-1*α*, and ATP5D in myocardium after drug treatment.^##^*P* < 0.01, ^#^*P* < 0.01, model group vs. sham group; ^∗∗^*P* < 0.01, ^∗^*P* < 0.05, treated group vs. model group.

In order to further confirm the regulatory effect of NXT on energy metabolism, the protein levels of SIRT1, PGC-1*α*, and ATP5D were measured by western blotting. The results are shown in Figure 10. Myocardial infarction led to a decrease in the protein levels of SIRT1, PGC-1*α*, and ATP5D in cardiac muscle tissue. Conversely, NXT reversed the reduction in the levels of these three proteins after myocardial infarction. In conclusion, NXT can enhance ATP synthesis, regulate energy metabolism and mitochondrial biosynthesis by enhancing the expression of SIRT1, PGC-1*α*, and ATP5D during myocardial infarction and further exert its protective effect on ischemic myocardial tissue.

## 4. Conclusion

A targeted analytical method for detecting 29 metabolites related to the energy metabolism in the cardiac muscle tissue was developed and used to investigate the mechanisms of rat acute myocardial infarction and the therapeutic effects of NXT.

Prior to the targeted metabolic analysis, a rat myocardial infarction model was established by ligation of the left anterior descending coronary artery and used to validate the protective effects of NXT. NXT demonstrated an effective cardioprotective effect against myocardial infarction based on the evaluation of cardiac function (LVEF and LVFS), ventricular structure indicators of the echocardiograms, and myocardial enzymes (cTnT). Furthermore, HE and Masson staining results suggested that NXT can decrease inflammatory infiltration, improve myocardial fibrosis, and reduce the infarct size in rats with myocardial infarction.

The 29 metabolites in the penumbral tissue of the left heart were accurately and quantitatively analyzed using the rat myocardial infarction model. Myocardial ischemia induced significant changes in the levels of 17 metabolites from different energy metabolic pathways, including four compounds in glycolysis metabolism, four compounds in TCA, three compounds in oxidative phosphorylation, four compounds in purine metabolism, and two compounds in glutathione metabolism, compared to the sham group. However, these perturbations could be reversed by NXT intervention, suggesting that the therapeutic effects of NXT on myocardial ischemia were caused in part by interferences with energy metabolisms. Furthermore, the expression of three proteins, SIRT1, PGC-1*α*, and ATP5D, regulating energy metabolism were detected. The results have showed that after myocardial infarction, the three proteins changed abnormally and reversed by NXT. These findings, in particular, shed light on the relationship between changes in metabolites from different energy metabolic pathways, energy metabolism–related proteins, and the mechanism of myocardial ischemia.

In summary, developed analytical methods provide potential tools for further disease-related studies. Moreover, the results of this study may aid in the investigation of the mechanism of myocardial ischemia, as well as the discovery of potential targets for NXT.

## Figures and Tables

**Table 1:** Data of echocardiographic left ventricular function in each group. ^##^*P* < 0.01, ^#^*P* < 0.01, model group vs. sham group; ^∗∗^*P* < 0.01, ^∗^*P* < 0.05, treatment group vs. model group.

Groups	Heart rate BPM	LVAW; s mm	LVAW; d mm	LVPW; s mm	LVPW; d mm	Cardiac output mL/min
Sham	365.73 ± 24.32	3.52 ± 0.55	1.97 ± 0.31	3.43 ± 0.42	2.30 ± 0.37	97.31 ± 15.01
Model	324.50 ± 16.18^##^	1.15 ± 0.12^##^	1.17 ± 0.16^##^	2.89 ± 0.52^#^	1.89 ± 0.22^#^	71.09 ± 12.21^##^
NXT-L	340.98 ± 14.29^∗^	2.09 ± 0.83^∗∗^	1.42 ± 0.24^∗^	3.25 ± 0.32	1.93 ± 0.12	79.39 ± 4.21^∗^
NXT-H	350.75 ± 14.61∗	2.30 ± 1.11^∗∗^	1.59 ± 0.45^∗^	3.27 ± 0.09^∗^	2.13 ± 0.18^∗^	81.10 ± 10.06^∗^
Val	336.98 ± 18.39	1.89 ± 0.90^∗^	1.49 ± 0.35^∗^	3.10 ± 0.07	1.93 ± 0.13^∗^	88.03 ± 11.15^∗^

**Table 2:** List of the 29 tested energy-related metabolites.

No.	Metabolite	Formula	Retention time (min)	Parent (m/z)	Daughter (m/z)	Declustering potential (V)	Collision energy (V)	Linear range (*μ*mol/L)	*R* ^2^
1	Pyruvate	C_3_H_3_O_3_^−^	1.36	87.1	43.1	-50	-12	25-500	0.9993
2	Lactate	C_3_H_5_O_3_^−^	1.82	89.2	43.0	-120	-15	10-500	0.9994
3	Cyclic AMP	C_10_H_12_N_5_O_6_P	1.96	328.0	134.0	-100	-30	0.1-5	0.9992
4	Alpha-ketoglutarate	C_5_H_6_O_5_	3.82	145.2	101.1	-50	-10	0.1-500	0.9986
5	Fumarate	C_4_H_2_O_4_^−2^	4.06	115.1	71.1	-50	-10	5-500	0.9990
6	Oxaloacetate	C_4_H_4_O_5_	4.41	131.1	87.1	-20	-18	2.5-250	0.9993
7	Succinate	C_4_H_4_O_4_^2-^	4.58	117.1	73.0	-47	-16	0.1-250	0.9987
8	L-malic acid	C_4_H_6_O_5_	4.99	133.0	115.1	-42	-14	0.1-100	0.9993
9	Flavin mononucleotide (FMN)	C_17_H_18_N_4_O_9_P^−3^	5.08	455.1	97.0	-100	-30	0.1-50	0.9993
10	Acetyl coenzyme A (acetyl-CoA)	C_23_H_38_N_7_O_17_P_3_S	5.81	808.2	408.1	-120	-47	0.1-25	0.9992
11	Adenosine monophosphate	C_10_H_14_N_5_O_7_P	6.17	346.1	79.0	-80	-60	10-500	0.9993
12	Dihydroxyacetone phosphate	C_3_H_7_O_6_P	6.21	169.1	97.0	-40	-16	5-500	0.9995
13	NAD	C_21_H_27_N_7_O_14_P_2_	6.39	662.1	540.0	-100	-20	0.1-25	0.9992
14	Cis-aconitate	C_6_H_6_0_6_	6.79	173.1	85.1	-45	-16	0.1-5	0.9991
15	Beta-D-fructose 6-phosphate	C_6_H_13_O_9_P	7.30	259.1	97.0	-50	-18	0.1-500	0.9990
16	ADP	C_10_H_15_N_5_O_10_P_2_	7.38	426.0	79.0	-100	-76	10-500	0.9985
17	Phosphoenolpyruvate	C_3_H_5_O_6_P	7.43	167.1	79.0	-50	-18	1-250	0.9990
18	Isocitrate	C_6_H_8_O_7_	7.49	191.0	73.0	-50	-30	0.1-10	0.9983
19	Citrate	C_6_H_5_O_7_^−3^	7.58	191.1	87.1	-50	-21	5-500	0.9992
20	3-Phospho-D-glycerate	C_3_H_7_O_7_P	7.75	185.0	97.0	-45	-21	10-500	0.9992
21	GMP	C_10_H_14_N_5_O_8_P	7.76	362.1	79.1	-100	-60	0.1-50	0.9821
22	D-glucose 6-phosphate	C_6_H_13_O_9_P	8.22	259.0	97.0	-45	-20	0.1-500	0.9995
23	Adenosine 5′-triphosphate (ATP)	C_10_H_16_N_5_O_13_P_3_	8.44	506.0	159.0	-100	-42	10-500	0.9993
24	Guanosine 5′-diphosphate (GDP)	C_10_H_15_N_5_O_11_P_2_	8.96	442.0	79.0	-100	-95	0.25-100	0.9993
25	Thiamine pyrophosphate (TPP)	C_12_H_19_ClN_4_O_7_P_2_S	9.34	423.0	302.0	-60	-20	0.1-250	0.9991
26	NADPH	C_21_H_30_N_7_O_17_P_3_	9.89	744.1	408.0	-40	-45	5-500	0.9990
27	NADP	C_21_H_29_N_7_O_17_P_3_+	9.89	742.1	620.0	-90	-25	0.25-250	0.9994
28	Guanosine 5′-triphosphate (GTP)	C_10_H_16_N_5_O_14_P_3_	10.02	522.0	159.1	-140	-34	1-100	0.9991
29	D-fructose 1,6-bisphosphate	C_6_H_14_O_12_P_2_	10.77	339.1	97.0	-50	-23	2.5-100	0.9991
IS	Succinic acid	C_4_H_6_O_4_		123.1	79.0	-47	-16		

## Data Availability

If the article is acceptable, the author will upload the original data supporting the research results in the form of a file.
